# Editorial: Robotics in the performance, safety and learning of surgery - what next?

**DOI:** 10.3389/frobt.2026.1927781

**Published:** 2026-09-02

**Authors:** Sanju Lama, Hani J. Marcus, Garnette R. Sutherland

**Affiliations:** 1 Project neuroArm Medical Robotics Program, Department of Clinical Neurosciences, University of Calgary, Calgary, AB, Canada; 2 Division of Neurosurgery, UCL Queen Square Institute of Neurology, London, United Kingdom

**Keywords:** AI, digital intelligence, IoT, internet of things, OR, robotics, standardization, surgery

## Introduction

As surgery has evolved over time through the imitation of art and the development of techniques and tools, the learning and performance of surgery have also advanced through the transfer of skills, knowledge, and principles through scholarly mentorship and apprenticeship. Guided by the underlying adage “*first do no harm”*, surgical safety remains a commitment to patients that cannot be emphasized enough.

Since the advent of imaging technologies, intra-operative monitoring, real-time image guidance, and robotics in the operating room (OR) at various times in history, our reliance on these technologies and digital platforms, including electronic medical records, has become intuitive and necessary. These adjuncts not only aid in lesion localization and the execution of precise and accurate care at the tool-tissue level, but they also generate a plethora of data in the often *closed-door and secure* environment of the OR, providing opportunities for quantifiable metrics. This was in part the theme behind this Research Topic … i.e., *how much has changed in the landscape of robot-assisted surgery and where are we heading next?*

The uptake (or lack thereof) of the widespread adoption of robotics in surgery and healthcare at large is incomplete without the mention of the da Vinci system (Intuitive Surgical, Sunnyvale, CA, United States), arguably the most successful commercially available system globally (Ricciardi et al., 2025). Designed and built for minimally invasive surgery, the system continues to evolve by incorporating a haptic interface or improving its already superb vision and tool maneuverability (endo-wrist-enabled dexterity) at the surgeon-machine interface.

Clinically integrated in the OR, pending commercialization, the neuroArm system (Project neuroArm, University of Calgary, AB, Canada), built in collaboration with Macdonald Dettwiller and Associates (MDA Space, Brampton, ON, Canada), happens to be the world’s first image-guided, MR-compatible robot for brain surgery. It is also a tele-capable system. Recreating the sight, sound, and touch of surgery at the sensory-immersive workstation (Sutherland et al., 2013) was a key focus of this innovation. The finesse of microsurgery and quantifying such being paramount, advanced sensors at the tool-tissue interface for transmitting haptic sensations to the surgeon at the workstation was a priority. Force scaling, motion, and 3D stereoscopic vision of the surgical site, along with robot kinematics stored in its memory and levels of redundancy for safety, enabled the digitization of surgery in real time. Despite these advances and perhaps given the complexity of engineering and clinical translation processes, robotics in general appears to encounter barriers or a lack of widespread uptake. *Are the barriers cost, OR workflow, safety considerations, machine factors, or human factors?*

In parallel, the evolution of advanced digital platforms, sensors, monitoring, and cloud-linked IoT systems and AI is rapid at the consumer level. However, their entry into the OR at the procedural level is somewhat nascent (Lama et al., 2026). Recent releases and uptake of generative AI may have influenced health administration, workflow, electronic charting, and disease interrogation for information and learning; however, the utility of AI or the notion of autonomous microsurgery is still somewhat remote (Fard et al., 2026).

Is it time, then, to revisit the role of machine advances with digital innovation, AI, and automation in robotic systems for surgery? How do these advances influence the performance, safety, and learning to create a quantifiable and standardizable paradigm in surgical care? With the principles of Industry 4.0 reshaping the world, could the OR and surgery be the next frontier? This would require a pragmatic, scientific, and clinical consideration across surgical subspecialties (Marcus et al., 2024). The contributions in this Research Topic attempt to highlight this evolving landscape. *What is possible and feasible? What next? What will be the next big disruption in the OR?*

Minimally invasive procedures are a key motivation and driver of robot-assisted surgery. Zhiyun et al. demonstrated through a case-control series using the TiRobot ForcePro Superior system that robotic screw implantation is associated with significantly reduced intraoperative blood loss, shorter incision lengths, alleviated pain, and better recovery of shoulder joint function. Regarding workflow and the integration of robotics in the OR, the accuracy of screw placement, OR time, length of hospital stays, and post-operative complications were found to be comparable between robot-assisted and conventional surgery.

Challenging the perception that robotic systems lack flexibility, Fritsch and Overschmidt presented an algorithmic framework for real-time configuration to a target pose for a hyper-redundant robotic end-effector. In an era where mathematical modeling and simulation offer close real-world representations, this novel inverse kinematic model suggests a potential pathway toward scalable, multi-joint, and multipurpose dexterous robotic end-effectors.

Virtual reality (VR) simulations in robotics continue to be an area of interest, with their importance explored and often established in the learning/training of surgeons in a risk-free environment. Kawashima and colleagues used an early non-inferior head-mounted VR simulation for a robot-assisted suturing task as opposed to a conventional console-based simulation. With early signals suggesting faster learning toward proficiency among the VR simulation group, further work and validation are needed to help establish its significance and potential for efficient training paradigms.

In the constrained anatomy of dental procedures where high-volume care is expected, Thieringer et al. explored the utility of digital planning systems tailored to patient-specific problems. While ongoing advances in the digital infrastructure of robotic platforms with iterative improvements may enable real-world integration, this work highlights the inherent challenges of translating concepts into routine practices and their implications for workflow.

In neurosurgery, accurate target localization is fundamental to surgical planning and execution. Using established neuronavigation software within a miniature robotic unit, Stealth AutoGuide TM (Medtronic, United States), Barth et al. examined the value of learning curve and workflow optimization in stereotactic biopsy procedures. The findings show promising levels of surgeon-independent accuracy and relatively seamless integration into the OR workflow, including procedural safety for biopsy. These results are indicative of the continued maturation of procedure-specific robotic units with built-in navigation capabilities.

A discussion of surgical robotics would be incomplete without reference to endoscopy, both for its minimal invasiveness and potential to recreate an algorithmic advantage over traditional systems. Through the use of interchangeable, articulated robotic end-effectors in an endoscopic, trans-nasal approach, Dimitrakakis and colleagues overcame the limitations of the current, conventional endoscopic toolset. Although it is pre-clinical, their study demonstrates improved operative access and surgeon dexterity, marking a viable proposition for extending robotic systems to include endoscopy.

## Intelligent surgical robotics as an equalizer

While this small Research Topic highlights the work of our peers and robot enthusiasts who are driving the integration of robotics into surgery, the devices in this Research Topic, along with the surgical team, add to the ever-expanding, data-rich environment of the OR, which is underutilized for digital innovation and appears to be a closed door for health data safety and compliance. Drawing inspiration from the aerospace industry, where machine precision, digital interconnectivity, quantification, standardization, and automation are deeply embedded, surgery continues to rely on human operators. Despite textbook knowledge and prolonged training for proficiency, variability is an unavoidable reality.

Robotics and AI offer an opportunity to address this variability. Present-day robotics not only lacks the finesse and flexibility required for microsurgery but also the structured and integrated digital intelligence necessary to mimic human expertise, experience, and judgment. When merged with the memory, high-dimensional computation, and predictive algorithms of AI, the formation of an ideal human-robot partnership is conceivable. Autonomous microsurgery would then be an achievable proposition.

For this to be realized, robust and scalable digital infrastructure is imperative that incorporates transparent AI, explainability, traceability, and the validity of digital signatures within the system. Post-quantum-level cybersecurity offers promise in the digital interplay of machine-to-machine authentication(Riva-Cambrin et al., 2026). At the same time, the rapid release of new algorithms and open-source platforms suggests that increasingly adaptive, agile, and interconnected robotic systems are within reach–a necessary equalizer and the next disruptor in the OR. When controlled for cost, affordability, and scalability, a broader adoption of robotics is inevitable–a paradigm for quantifiable and standardizable surgery and a necessary antidote to human variability.

In the complex and dynamic landscape of surgery and robotics, its knowledge and predictive autonomy may very well help level the playing field while empowering discoveries and innovations in perpetuity.
